# Supplementary material for: Premovement Suppression of Corticospinal Excitability Is Modulated by Reaction Time Task Requirements
Source: Eur J Neurosci. 2026 Aug 23;64(4):e70659. doi: 10.1111/ejn.70659 (PMC13500203; doi:10.1111/ejn.70659)
Supplement: Supplementary file 1 [file EJN-64-0-s001.pdf]

Supplementary Analysis: MEP amplitude at baseline vs. -2500 ms

To examine whether the -2500 ms TMS time point was reflective of an out-of-task resting activation level, integrated MEP amplitude was compared between TMS applied just after determination of resting motor threshold (i.e. resting baseline, measured in subset of 12 participants) and when TMS was applied at -2500 ms with respect to the go-signal in each of the three RT conditions. These values were compared using a generalized linear mixed effects model with task condition (4 levels: Resting Baseline, simple RT, choice RT, go/no-go RT) as a fixed factor. Random intercepts were specified for participants, and the model used a Gamma distribution for the residuals with a log link function (e.g., MEP amplitude ~ TaskCondition + (1 | Participant), family = Gamma(link="log")).

Supplementary Results: MEP amplitude at baseline vs. -2500 ms

Integrated MEP amplitude as a function of task condition is presented in Figure S1. Integrated MEP amplitude when TMS was applied at resting baseline was compared to MEP amplitude when TMS was applied 2500 ms prior to the go-signal in each of the RT tasks. Analysis showed a significant effect of condition, χ^2 (3) = 187.20, $p < .001$, with post-hoc comparisons indicating that while MEP amplitude was significantly larger in all three RT tasks compared to baseline (all p-values < .001), there was no difference in amplitude between the RT tasks (all p-values > .229). This result indicates that corticospinal activation was significantly higher at the -2500 ms time point that it was during a true out-of-task resting state.

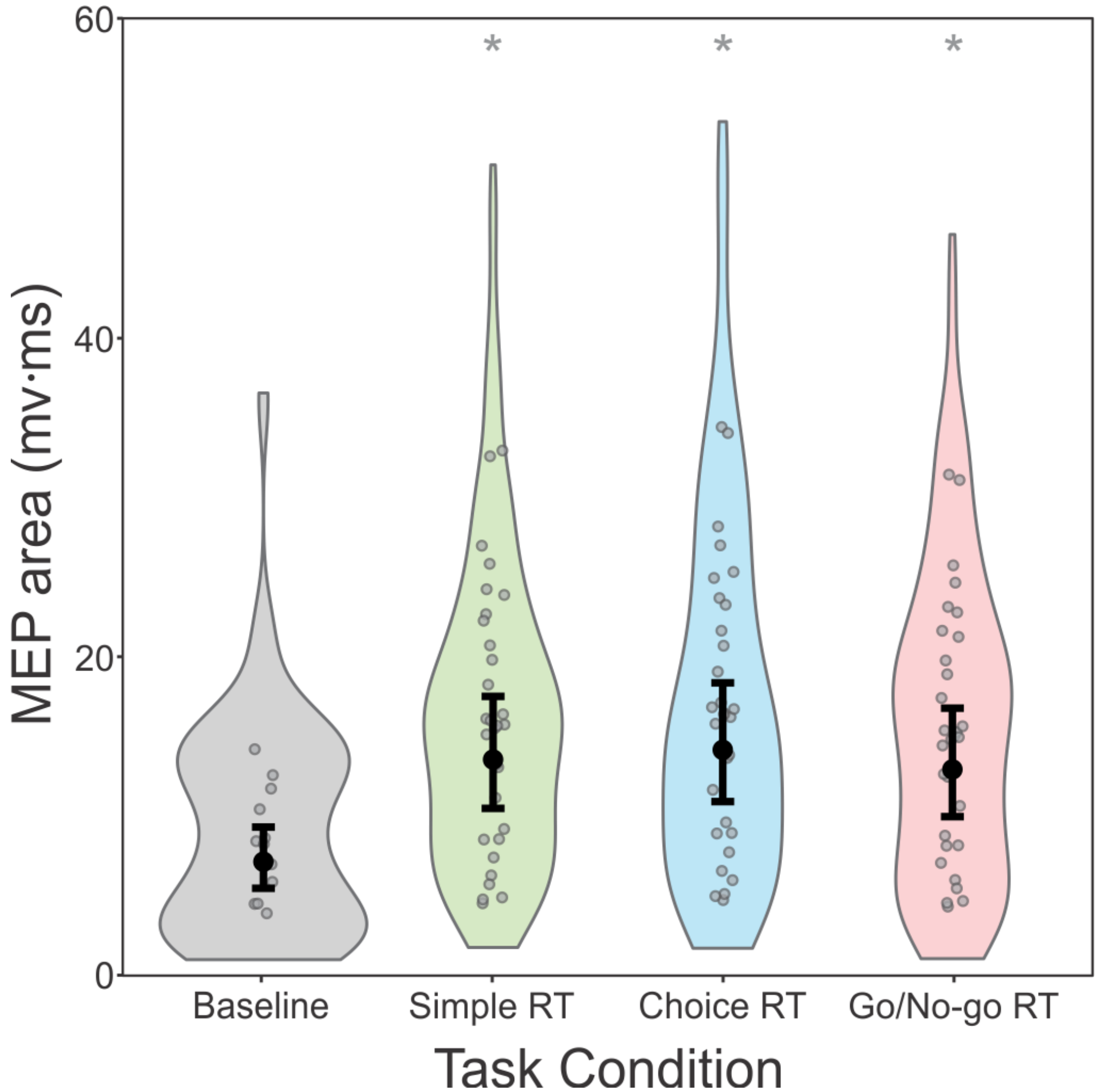

Figure S1. Motor-evoked potential (MEP) integrated area at baseline (resting; grey), and at the -2500 ms TMS application time for each reaction time (RT) task (green = simple RT; blue = choice RT; red = go/no-go RT). Fixed effect means and 95% CI are shown as large markers and error bars, individual subject estimated mean MEP amplitudes are shown as small grey markers, and violin plots represent the distribution of raw data, Means are back-transformed from the log scale. Note that MEP amplitude was significantly (*) larger for all three RT tasks at the -2500 ms TMS application time compared to baseline.

Supplementary Analysis: MEP amplitude with inclusion of -2500 ms TMS time point

To investigate how corticospinal excitability evolved as the go-signal approached (with inclusion of the -2500 ms TMS time point), MEP amplitude in the wrist extensor was analyzed using a generalized linear mixed effects model with interacting fixed factors of

TMS time prior to the go-signal (7 levels: −2500, −500, −300, −150, −100, −50, 0) and RT task (3 levels: simple RT, choice RT, go/no-go RT). Random intercepts were specified for participants, and the model used a Gamma distribution for the residuals with a log link function (e.g., MEP amplitude ~ TMStime * Task + (1 | Participant), family = Gamma(link="log")).

Supplementary Results: MEP amplitude with inclusion of -2500 ms TMS time point

Integrated MEP amplitude as a function of TMS time during each RT task is presented in Figure S2. Significant main effects were found for TMS time, χ^2 (6) = 452.869, $p < .001$, and RT task, χ^2 (2) = 25.222, $p < .001$, as well as a marginally significant interaction between TMS time and task, χ^2 (12) = 20.501, $p = .058$. Post-hoc tests indicated that in each RT task, MEP amplitude was significantly smaller (all p-values < .01) when TMS was applied at -150 ms, -100 ms, -50 ms, and at the go-signal, as compared to when TMS was applied at -2500 ms, -500 ms and -300 ms. Furthermore, in the Go/No-go RT task, MEP amplitude was significantly smaller at -50 ms and at the go-signal compared to -150 ms (p-values < .002). When comparing between RT tasks at each TMS time point, MEP amplitude for the choice RT task was significantly larger than that for the go/no-go RT task ($p < .001$) when TMS was applied either 50 ms before or coincident with the go-signal. Similarly, MEP amplitude for the choice RT task was significantly larger than that for the simple RT task when TMS was applied coincident with the go-signal ($p = .007$) but did not reach significance for the 50 ms time point ($p = .082$). These results show that the inclusion of the -2500 ms TMS time point do not materially change the results or conclusions of the study.

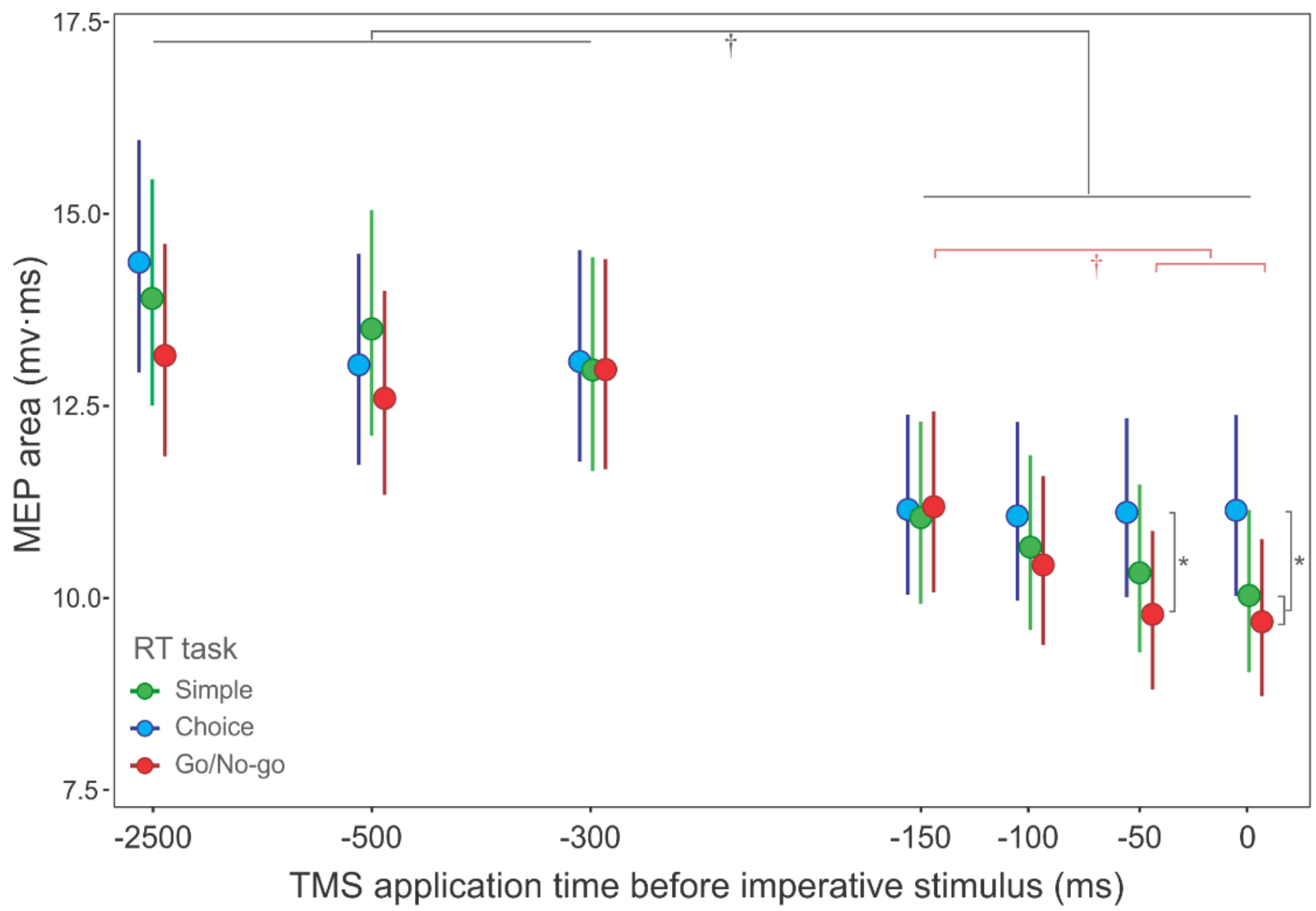

Figure S2. Motor-evoked potential (MEP) integrated area as a function of TMS stimulation time for each reaction time (RT) task (green = simple RT; blue = choice RT; red = go/no-go RT), showing fixed effect means and 95% CI for each task. Note that means are back-transformed from the log scale. Asterisk (*) indicates significant difference between tasks at each time point. Dagger (†) indicates significant difference between time points (grey = all tasks, red = go/no-go only).
